# The impact of co-expression of wild-type EGFR and its ligands determined by immunohistochemistry for response to treatment with cetuximab in patients with metastatic colorectal cancer

**DOI:** 10.18632/oncotarget.13835

**Published:** 2016-12-09

**Authors:** Said Khelwatty, Sharadah Essapen, Izhar Bagwan, Margaret Green, Alan Seddon, Helmout Modjtahedi

**Affiliations:** ^1^ School of Life Sciences, Pharmacy and Chemistry, Kingston University London, Kingston UK; ^2^ St Luke's Cancer Centre, Royal Surrey County Hospital, Guildford, UK; ^3^ Department of Histopathology, Royal Surrey County Hospital, Guildford, UK

**Keywords:** colorectal cancer, EGFR, immunohistochemistry, cetuximab, response

## Abstract

Anti-EGFR mAbs cetuximab and panitumumab are routinely used for the treatment of patients with KRAS-wild type metastatic colorectal cancer (mCRC). However, in some patients their efficacy remains modest and with no clear association between the EGFR protein expression determined by PharmDx™ kit, and response to anti-EGFR therapies. Therefore, we investigated the relative expression and predictive value of wild-type EGFR (wtEGFR), mutated EGFRvIII and EGFR ligand proteins in mCRC patients treated with cetuximab. The expression levels of wtEGFR, EGFRvIII, and EGFR ligand were determined by immunohistochemistry (IHC) in 60 tumour specimens using specific antibodies. Sections were scored according to the percentage of positive tumour cells, intensity and cellular location of staining, and these were associated with response, overall survival (OS) and progression-free survival (PFS). At cut-off value > 5%, wtEGFR, and EGFRvIII were present in 44%, and 41%, betacellulin (BTC) in 72%, followed by epigen (67%), TGFα (58%), amphiregulin (34%), EGF (31%) of the cases, respectively and 96% of the wtEGFR positive cases had co-expression of at least one ligand. We found a significant association between the expression of wtEGFR and poor response to cetuximab. In addition, the co-expression of wtEGFR with one ligand at a cut-off value of > 5% and > 10% was associated with worse response to cetuximab (*P* = 0.021, and *P* = 0.005 respectively). We found a 3-fold and 5-fold increased risk of shorter OS with expression of BTC and epigen. Interestingly, the expression of wtEGFR and its co-expression with one or two ligands was associated with shorter PFS but not with OS. The relative expression of wtEGFR and its competing ligands, which is the target for therapeutic interventions with anti-EGFR antibodies, could serve as a more reliable predictive biomarker of response to therapy with anti-EGFR mAbs in mCRC patients and warrants further investigation in large prospective studies.

## INTRODUCTION

Colorectal cancer remains one of the leading causes of cancer deaths worldwide [1]. In 2016, colorectal cancer is estimated to be the third most commonly diagnosed cancer (134,900) and the third leading cause of cancer deaths (49,190) in the USA [2], highlighting the need for developing more effective and less toxic therapeutic agents. In the last three decades, the aberrant expression of the epidermal growth factor receptor (EGFR) has been reported in a wide range of tumours including colorectal, head and neck and lung cancers and the EGFR is currently an important therapeutic target for targeted therapy with anti-EGFR antibodies in such patients [3]. While treatment with a combination of antibodies and cytotoxic drugs improves response rate and median time to progression in some colorectal cancer patients, the duration of response is often limited in most patients with an advanced stage of the disease [3]. To date, of the anti-EGFR monoclonal antibodies (mAbs), only cetuximab and panitumumab have been approved for the treatment of patients with metastatic colorectal cancer (mCRC) with RAS wild-type status [4–8]. However, no clear association has been found between the expression level of the EGFR protein in the tumours, determined by the FDA approved EGFR PharmDx™ kit, or other standard anti-EGFR antibodies, and the response to the EGFR inhibitors [3, 9–12]. We have previously shown that kits such as the EGFR PharmDx™, does not discriminate between the wtEGFR and EGFRvIII, and as such could have a major contribution to the lack of association between the expression of EGFR and response to anti-EGFR antibodies in such studies [13]. This is of vital importance as the wtEGFR protein is not only the therapeutic target for anti-EGFR antibodies but also transmits the mitogenic action of competing autocrine and paracrine EGFR ligands [14].

In recent years, while RAS mutation has served as an important negative predictive biomarker for response to therapy with anti-EGFR mAbs in patients, not all patients with wild type *KRAS* gain benefit from therapy with anti-EGFR mAbs [15, 16] and objective responses of up to 44% have been reported in mCRC patients with *KRAS* mutations treated with cetuximab in other studies [17]. In addition, several studies of gene expression of some of the EGFR ligands have indicated that an increased expression of amphiregulin, epiregulin, TGFα may act as prognostic indicators and predictive biomarkers of response to therapy with anti-EGFR mAbs [18–25]. However, to our knowledge; there have been no comprehensive studies on the protein expression levels of wtEGFR and all EGFR ligands in mCRC patients and their predictive values for response to treatment with anti-EGFR mAbs such as cetuximab. Therefore, in this study, using specific antibodies we have investigated the expression levels of wtEGFR, EGFRvIII, phosphorylated EGFR and all seven EGFR ligands in tumour specimens from 60 mCRC patients with *KRAS* wild-type status treated with cetuximab and their associations with clinicopathological parameters, PFS and OS.

## RESULTS

### Clinicopathological features

Patient clinicopathological characteristics are summarised in Table 1. The median patient follow-up time was 4 years, median OS was 2.7 years and median PFS was 3 months. All patients received FOLFIRI (irinotecan and modified de Gramont) plus cetuximab or FOLFOX (oxaliplatin and modified de Gramont) plus cetuximab therapies as first line chemotherapy. An improved OS (*P = 0.017*) and a longer PFS was observed in patients with vascular invasion, which is unusual. Those patients with T4 cancers had a significantly shorter PFS (*P = 0.014*), compared with those cancers with a less advanced T stage (Table 1).

**Table 1:** Clinicopathological parameters and survival of 60 mCRC patients treated with anti-EGFR mAb cetuximab

Characteristics	Number of patients (%)	OS in years (mean ± SE)	95% CI	*P*-value	PFS in months (mean ± SE)	95% CI	*P*-value
**Age in years**							
**≤ 70**	44 (73)	4.8 ± 0.5	3.2–4.7		17.5 ± 4.3	9.0–25.9	
**> 70**	16 (27)	4.1 ± 0.4	3.8–5.6	*NS*	9.2 ± 2.4	4.4–14.0	*NS*
**Gender**							
**Male**	42 (70)	4.9 ± 0.5	3.9–6.0		15.6 ± 4.2	7.4–23.9	
**Female**	18 (30)	3.9 ± 0.3	3.3–4.5	*NS*	13.8 ± 3.9	6.2–21.4	*NS*
**Tumour Site**							
**Right colon**	11 (18)	3.9 ± 0.0	3.9–3.9		7.9 ± 4.4	0.0–16.5	
**Left colon**	29 (48)	3.8 ± 0.2	3.4–4.2		12.3 ± 2.4	7.6–16.9	
**Liver Resections**	14 (23)	3.7 ± 0.3	3.0–4.4	*NS*	10.6 ± 3.5	3.8–17.4	*NS*
T stage*							
**< T4**	16 (27)	3.6 ± 0.2	3.2–4.0		16.1 ± 3.6	8.9–23.1	
**T4**	23 (38)	4.2 ± 0.4	3.4–5.0	*NS*	7.4 ± 2.2	3.0–11.7	***0.014***
N Stage*							
**< N2**	18 (30)	3.8 ± 0.3	3.3–4.3		12.1 ± 3.0	6.2–18.0	
**N2**	21 (35)	3.7 ± 0.2	3.3–4.2	*NS*	10.7 ± 2.9	4.9–16.5	*NS*
Vascular Invasion*							
**V0**	16 (26)	3.3 ±0.2	2.9–3.7		8.3 ± 2.9	2.6–14.0	
**V1**	23 (38)	4.1 ± 0.2	3.6–4.5	***0.017***	13.6 ± 2.8	8.0–19.1	*NS*
Grade*							
**< G3**	29 (48)	3.8 ± 0.2	3.4–4.2		12.2 ± 2.4	7.4–16.9	
**G3**	10 (16)	4.1 ± 0.2	3.7–4.4	*NS*	6.6 ± 3.7	0.0–14.0	*NS*
**Chemotherapy**							
**Folfiri + Cetuximab**	31 (51)	4.8 ± 0.5	3.8–5.9		11.6 ± 2.8	6.0–17.2	
**Folfox + Cetuximab**	29 (48)	4.4 ± 0.5	3.4–5.4	*NS*	18.6 ± 5.4	8.1–29.2	*NS*

OS and PFS relative to the indicated features were determined by Kaplan-Meier analysis and the log-rank test. *P*-value of *≤ 0.05* was considered significant.

* data for T stage, N stage, Vascular invasion, and grade missing in 21 patients. OS and PFS analysis was conducted by omitting the missing data.

### Immunohistochemical expression of EGFR, and EGFR ligands

**Figure 1:**
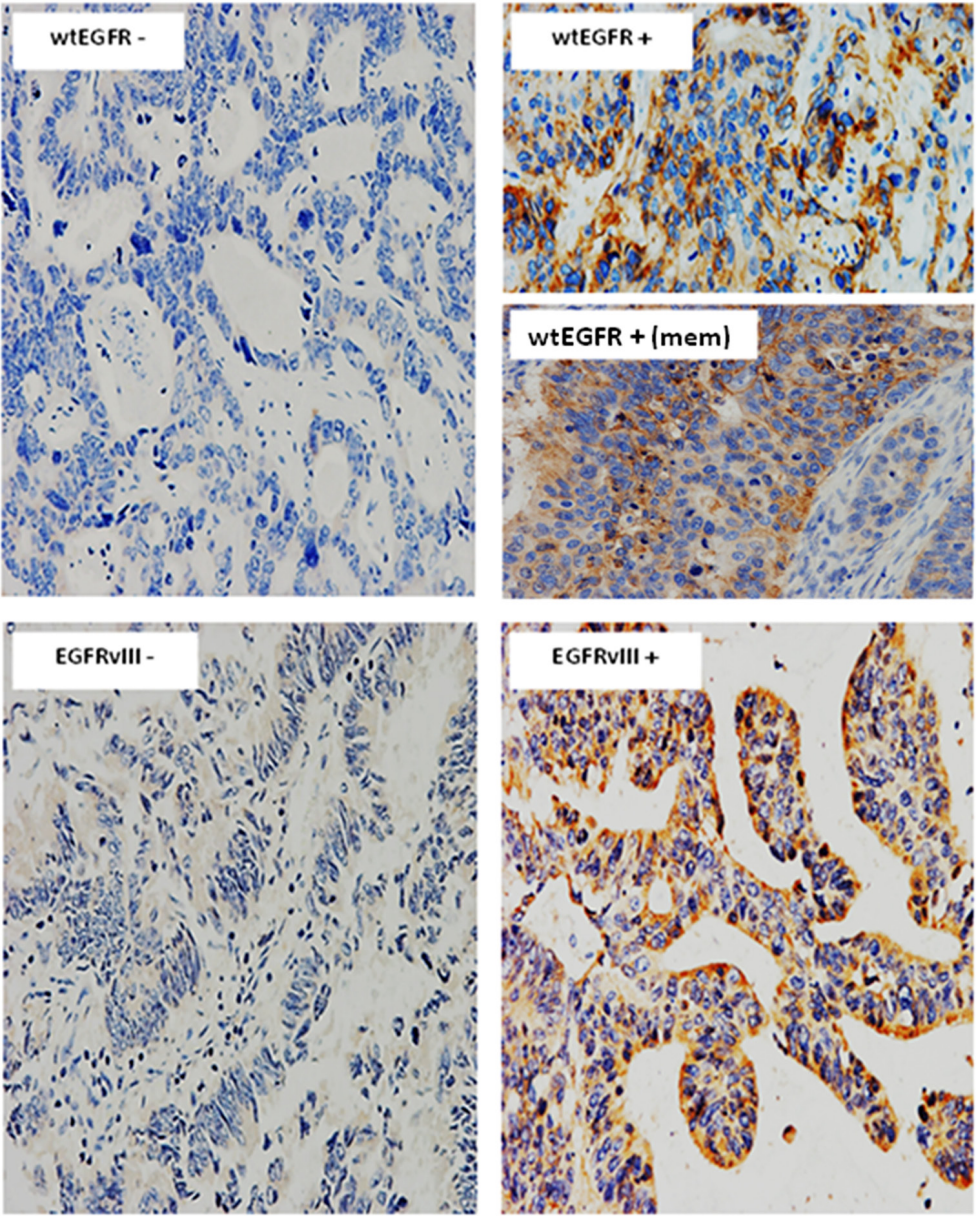
Immunostaining of wtEGFR and EGFRvIII in mCRC specimens Immunostaining of wtEGFR, and EGFRvIII in formalin fixed paraffin embedded tumour sections stained immunohistochemically, as described under methods and patients section. Magnification: x200

**Figure 2:**
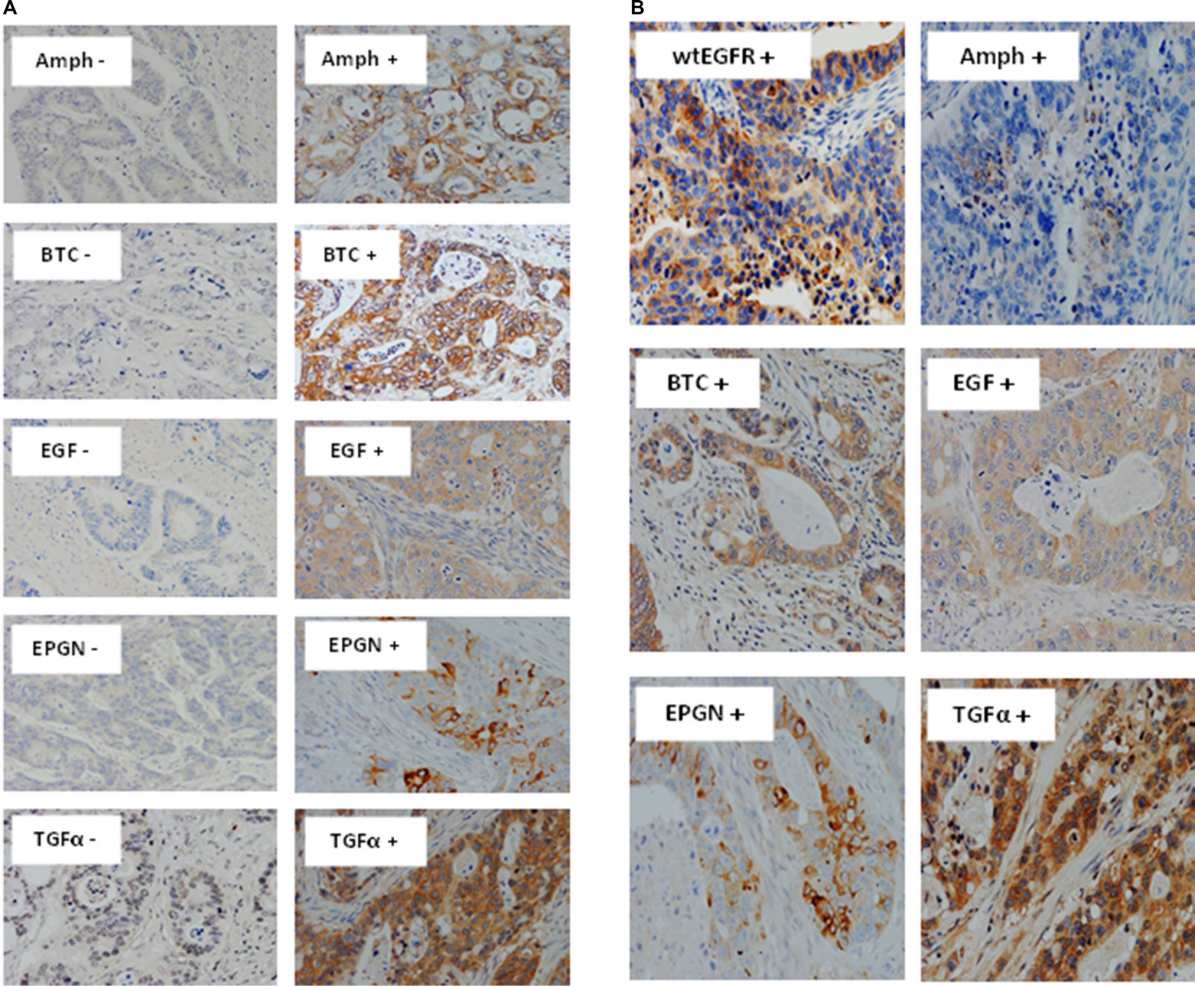
Immunostaining of EGFR ligands and co-expression with wtEGFR in mCRC specimens Expression of Amphiregulin, BTC, EGF, Epigen, and TGFα (**A**), and co-expression of wtEGFR, Amphiregulin, BTC, EGF, epigen, and TGFα in a particular patient (**B**) in formalin fixed paraffin embedded tumour sections stained immunohistochemically as described under methods and patients section. Magnification: x200

For the first time, in this study we determined the relative expression of EGFR using mAbs specific for the wild-type and EGFRvIII as well as the expression and co-expression of all EGFR ligands. Of the 60 cases examined, 44% and 41% were found to be positive for wtEGFR and EGFRvIII, respectively. The predominant staining pattern of wtEGFR was cytoplasmic (44%), with some cases having membranous staining (12%), while EGFRvIII expression was only cytoplasmic (Figure 1, Table 2). Of the EGFR ligands, BTC was the most commonly expressed ligand (72%), followed by epigen (67%), TGFα (58%), amphiregulin (34%), EGF (31%) (Figure 2A, Table 2). The expression of HB-EGF, epiregulin, and phosphorylated EGFR (1068 and 1173) were undetectable in tumour sections in this study. No nuclear staining was detected in this study.

**Table 2:** Immunohistochemical expression of EGFR and EGFR ligands and their co-expressions in 60 mCRC patients using the Fisher's exact test, FET

Variables	No. of positive tumours (%)								
	% Positive tumour cells				Intensity			Location	
	> 5	> 10	> 20	> 50	1+	2+	3+	Mem	Cyto
**wtEGFR**	27 (44)	23 (38)	16 (26)	8 (13)	27 (44)	1 (2)	0	7 (12)	27 (44)
**EGFRvIII**	25 (41)	25 (41)	17 (28)	12 (20)	23 (38)	1 (2)	1 (2)	0	25 (41)
**Amphiregulin**	21 (34)	16 (27)	7 (12)	5 (8)	18 (30)	3 (5)	0	0	21 (34)
**BTC**	43 (72)	40 (67)	34 (57)	19 (32)	18 (30)	29 (48)	1 (2)	0	43 (72)
**EGF**	19 (31)	17 (28)	12 (20)	7 (12)	20 (33)	1 (2)	0	0	19 (31)
**Epigen**	41 (67)	33 (54)	25 (41)	17 (28)	33 (54)	9 (15)	1 (2)	0	40 (66)
**TGFα**	35 (58)	33 (55)	30 (50)	26 (43)	31 (52)	11 (18)	0	0	35 (58)
**wtEGFR/Amph**	13 (22)	11 (18)	3 (5)	2 (3)	10 (17)	0	0	-	-
**wtEGFR/BTC**	18 (30)	13 (22)	8 (13)	2 (3)	6 (10)	0	0	-	-
**wtEGFR/EGF**	6 (10)	4 (7)	3 (5)	0	8 (13)	0	0	-	-
**wtEGFR/Epigen**	17 (28)	11 (18)	6 (10)	1 (2)	12 (20)	0	0	-	-
**wtEGFR/TGFα**	16 (27)	12 (20)	8 (13)	3 (5)	13 (22)	1 (2)	0	-	-
**wtEGFR/1 ligand**	26 (43)	22 (37)	15 (25)	7 (12)	24 (40)	1(2)	0	-	-
**wtEGFR/ 2 ligands**	21 (35)	17 (28)	8 (13)	6 (10)	16 (27)	0	0	-	-
**wtEGFR/ 3 ligands**	14 (23)	10 (17)	4 (7)	0	8 (13)	0	0	-	-
**wtEGFR/ 4 ligands**	9 (15)	2 (3)	0	0	1 (2)	0	0	-	-

Abbreviations: Mem, Membranous; Cyt, Cytoplasmic

*P*-value of ≤ 0.05 was considered significant

Co-expression of wtEGFR with any one, two, three or four ligands was 43%, 35%, 23% and 15% respectively (Table 2). In addition, one patient co-expressed wtEGFR with all five EGFR ligands (Figure 2B).

### Expression of wtEGFR and EGFR ligands and their association with response to therapy and disease progression

A significant association was found between wtEGFR expression at cut-off value of > 5% positive tumour cells and poor response to treatment with cetuximab (*P = 0.026*) as well as disease progression (*P = 0.002*) (Table 3). Of the EGFR ligands, the expression of amphiregulin at a cut-off value of > 10% tumour cells (*P = 0.013*), EGF (>50%) (*P = 0.045*), and co-expression of epigen/BTC (*P = 0.032*) were significantly associated with an increased disease progression in this study (Table 3), while BTC expression at cut-off value of > 5% was associated with decreased disease progression (*P = 0.006*).

**Table 3:** The association of wtEGFR and EGFR ligands at cut-off value of > 5% positive immunostaining with response to treatment with cetuximab and disease progression in 60 mCRC patients using the Fisher's exact test, FET

Variables					Association with:				
					Response to cetuximab				
			Yes		No			*P*-value	
**wtEGFR**	+ve		16		11			*0.026*	
	–ve		28		5				
**wtEGFR/1 ligand**	+ve		15		11			*0.021*	
	–ve		29		5				
**wtEGFR/1 ligand^§^**	+ve		11		11			*0.005*	
	–ve		33		5				
**wtEGFR/BTC^§^**	+ve		5		8			*0.003*	
	–ve		39		8				
**wtEGFR/BTC^†^**	+ve		3		5			*0.026*	
	–ve		41		11				
**wtEGFR/TGFα^§^**	+ve		5		7			*0.01*	
	–ve		39		9				

						Disease Progression		
				Yes		No	*P*-value	
**wtEGFR**		+ve		26		1	*0.002*	
		–ve		20		13		
**Amphiregulin^§^**		+ve		16		0	*0.013*	
		–ve		30		14		
**BTC**		+ve		29		14	*0.006*	
		–ve		17		0		
EGF*		+ve		3		4	*0.045*	
		–ve		43		10		
**Epigen/BTC**		+ve		20		11	*0.032*	
		–ve		26		3		
**wtEGFR/Amph**		+ve		13		0	*0.027*	
		–ve		33		14		
**wtEGFR/BTC**		+ve		17		1	*0.045*	
		–ve		29		13		
**wtEGFR/Epigen**		+ve		16		1	*0.05*	
		–ve		30		13		
**wtEGFR/TGFα**		+ve		16		0	*0.013*	
		–ve		30		14		
**wtEGFR/1 ligand**		+ve		25		1	*0.002*	
		–ve		21		13		
**wtEGFR/ 2 ligands**		+ve		20		1	*0.022*	
		–ve		26		13		
**wtEGFR/ 3 ligands**		+ve		14		0	*0.026*	
		–ve		32		14		

§, at cut-off value of > 10%; †, at cut-off value of > 20%; *, at cut-off value of > 50%

*P*-value of ≤ 0.05 was considered significant.

The co-expression of wtEGFR with one ligand at a cut-off value of >5% and > 10% was associated with worse response to cetuximab (*P = 0.021, and P = 0.005* respectively) (Table 3). This was also true in the case of the co-expression of wtEGFR with BTC at cut-off values of >10% and >20% and wtEGFR and TGFα at cut-off value of >10% (Table 3). Interestingly, the co-expressions of wtEGFR and amphiregulin, BTC, epigen, and TGFα were all significantly associated with a decreased disease progression (Table 3). A significant association was also found between the co-expression of wtEGFR with one ligand (*P = 0.002*), two ligands (*P = 0.022*) or, three ligands (*P = 0.026*) at cut-off value of >5% and disease progression in this study (Table 3).

### Impact of EGFR and EGFR ligands expression on overall survival

**Figure 3:**
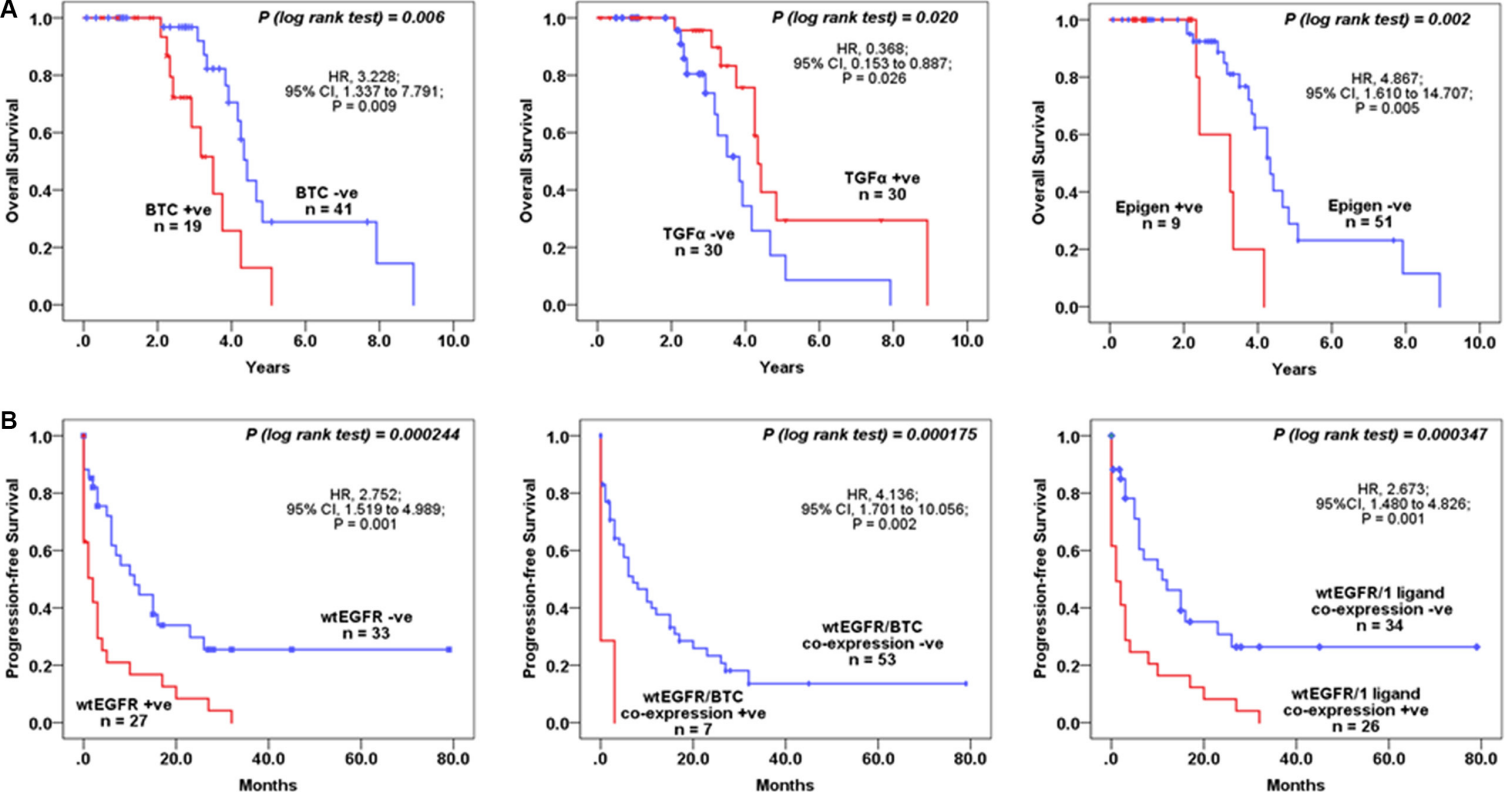
The association between wtEGFR and EGFR ligands and OS and PFS in mCRC patients treated with cetuximab Kaplan-Meier survival curves showing the impact on the OS of the patients with BTC, TGFα, and Epigen, expression (**A**), PFS with wtEGFR, co-expressions of wtEGFR and BTC, and wtEGFR and 1 ligand (**B**). A log-rank test value of *P ≤ 0.05* was considered statistical significance.

The association between the expression of wtEGFR and its ligands and OS was investigated using Kaplan-Meier curves and log rank-test. No significant associations were found between the expression of wtEGFR and EGFRvIII and OS in this study. BTC positivity in >50% of tumour cells (*P = 0.003*) and epigen expression with an intensity of 2+ (*P = 0.002*) were significantly associated with poorer OS (Figure 3A). Interestingly, the expression of TGFα was found to be significantly associated with a better OS (*P = 0.020*) (Figure 3A).

Using univariate analysis, we found a 3-fold and 5-fold increased risk of a shorter OS with expression of BTC (*P = 0.009*) and epigen (*P = 0.005*) (Table 4). BTC (*P = 0.023*), epigen (*P = 0.005*), and TGFα (*P = 0.016*) were all found to remain independent prognostic factors for survival when analysed using multivariate analysis in this study (Table 4).

**Table 4:** Univariate and multivariate analysis related to OS and PFS in 60 mCRC treated with anti-EGFR mAb cetuximab

Variables					Overall Survival (OS)							
			Univariate						Multivariate			
	**HR**		**95% CI**		***P-value***			**HR**	**95% CI**		***P-value***	
**Vascular Invasion**	0.201		0.048–0.834		*0.027*			0.204	0.049–0.852		*0.029*	
**BTC^†^**	3.228		1.337–7.791		*0.009*			4.463	1.235–16.133		*0.023*	
**Epigen (2+)**	4.867		1.610–14.707		*0.005*			11.533	2.092–63.569		*0.005*	
**TGFα (>20%)**	0.368		0.153–0.887		*0.026*			0.073	0.009–0.612		*0.016*	

						Progression-free Survival (PFS)						
				Univariate						Multivariate		
		HR		95% CI		*P-value*	HR			95% CI		*P-value*
**Tumour Stage**		0.442		0.202–0.966		*0.041*	0.351			0.146–0.844		*0.019*
**wtEGFR (> 5%)**		2.752		1.519–4.989		*0.001*	2.829			1.316–6.079		*0.008*
**Amphiregulin (2+)**		4.554		1.301–15.936		*0.018*	-			-		*NS*
**wtEGFR/1 ligand**		2.673		1.480–4.826		*0.001*	2.717			1.274–5.792		*0.010*
**wtEGFR/2 ligands**		2.141		1.187–3.862		*0.011*	2.306			1.093–4.862		*0.028*
wtEGFR/Epigen*		2.063		1.111–3.831		*0.022*	2.450			1.119–5.364		*0.025*
wtEGFR/BTC**		4.136		1.701–10.056		*0.002*	3.814			1.315–11.060		*0.014*

Abbreviations: HR, hazard ratio; CI, confidence interval; †, cut-off value > 50% used for OS and PFS; *, cut-off value of > 5% used; **, cut-off value of > 5% (wtEGFR) and > 50% (BTC); NS, Not significant

*P*-value of ≤ 0.05 was considered significant

### Impact of EGFR and EGFR ligands expression on progression-free survival

Interestingly, PFS was significantly shorter in patients with wtEGFR expression > 5% positive tumour cells (*P = 0.000244*), which in this study was found to be predominantly cytoplasmic (Figures 1 and 3B). In addition, using univariate and multivariate analyses we found that the expression of wtEGFR (> 5%) increased the risk of shorter PFS by nearly 3-fold (*P = 0.001*) and remain an independent predictive biomarker of shorter PFS (*P = 0.008*) (Table 4). The expression of amphiregulin was found to be significantly associated with a shorter PFS (*P = 0.004*) and increased the risk of a shorter PFS by nearly 5-fold (*P = 0.018*) (Table 4). However, in multivariate analysis this association failed to reach statistical significance (Table 4). Interestingly, the co-expression of wtEGFR and any one (*P = 0.000347*) or two (*P = 0.006*) ligands was found to be significantly associated with shorter PFS in this study (Table 4, Figure 3B). Of these, the co-expression of wtEGFR and epigen at cut-off value of >5% was found to be significantly associated with shorter PFS (*P = 0.013*) in both univariate (*P = 0.022*) and multivariate analyses (*P = 0.025*). In addition, the co-expression of wtEGFR (> 5%) and BTC (> 50%) was significantly associated with shorter PFS (*P = 0.000175*) (Table 4, Figure 3B). In univariate analysis, this association was shown to increase the risk of shorter PFS by 4-fold (*P = 0.002*) and remained an independent predictive biomarker of shorter PFS (*P = 0.014*) (Table 4). Like OS, EGFRvIII was not significantly associated to PFS in this study (Data not shown).

## DISCUSSION

At present mCRC patients are selected for anti-EGFR mAb therapy provided their tumours do not harbour any RAS mutations, as no clear associations have yet been found between the expression of EGFR protein determined by immunohistochemistry and response to these inhibitors [4, 26–28]. A major contributing factor for such discordance could be the use of antibodies that do not discriminate between the wild-type and mutated forms of the EGFR in such patients [13]. Other contributing factors could be the use of very low cut-off value of EGFR positivity in the earlier studies (i.e. EGFR positivity when immunostaining is present in ≥1% of tumour cells) [29, 30], and discordance between the expression of EGFR in the primary tumour and the metastatic sites [31, 32]. While the mutational status of *KRAS* has been a well-known negative predictor of response to antibody therapy, the efficacy of anti-EGFR mAb therapy is modest even in some *KRAS*-wild type mCRC patients, and about 40% of patients without *KRAS* or *BRAF* mutations also have poor response to therapy with anti-EGFR antibodies [33]. In addition, objective responses have been detected in patients with *KRAS*-mutated tumours [17, 34]. Indeed, the latest American Society of Clinical Oncology provisional clinical opinion recommended that patients considered for anti-EGFR therapy should be tested for mutations in *KRAS* exons2 (codons 12 and 13), as well as extended *KRAS* exons 3 (codons 59 and 61) and 4 (codons 117 and 146) and *NRAS* exons 2 (codons 12 and 13), 3 (codons 59 and 61), and 4 (codons 117 and 146), as patients with such mutations are unlikely to benefit from therapy with anti-EGFR antibodies. While mutation testing to include *NRAS* in mCRC patients may improve the predictive value of such “passengers biomarker” for response to anti-EGFR mAb therapy, *NRAS* mutations are extremely rare and comprise only 2% of the mCRC patient population [4, 35]. Consequently, this present study was designed to investigate the relative expression and predictive value wtEGFR and EGFRvIII proteins and EGFR ligands, using specific antibodies in 60 *KRAS*-wild type mCRC patients treated with cetuximab.

We have shown previously that inconsistencies in the use of different cut-off values for EGFR immunostaining can be a major contributing factor for the variation in the expression and consequently the impact on the clinical outcome of anti-EGFR treatment in patients [36]. In this study we therefore evaluated the expression of EGFR and EGFR ligands using various cut-off values, as well as the location of the immunostaining (Table 2). We found the expression of wtEGFR, at cut-off value >5% positive tumour cells, to be a predictor of poor response to cetuximab, and an independent predictive biomarker of shorter PFS in this study. Interestingly, the predominant pattern of wtEGFR immunostaining in this study was cytoplasmic. Since, the binding site of anti-EGFR mAb cetuximab is on the extracellular domain of the EGFR, it is incapable of binding to the cytoplasmic EGFR and inducing the antibody-dependent cell-mediated cytotoxicity (ADCC) [37]. As a result, the association between cytoplasmic wtEGFR expression and poor response to cetuximab observed in our study is plausible. Further analysis of our data revealed that up to 40% of the tumours used in this study were treated with cetuximab prior to their surgical resection and 96% of wtEGFR positive cases co-expressed at least one EGFR ligand. One of the known mechanisms of action of cetuximab is downregulation of the EGFR by binding to the receptor and resulting in its internalisation. In addition, few preclinical data in other cancer models have reported that binding of EGFR ligands can also accelerate EGFR internalisation and as such elevate its localisation in the cytoplasm [38, 39]. It is therefore plausible that treatment with cetuximab and the abundant expression of one or more EGFR ligand could in fact have contributed to the downregulation of the EGFR from the cell surface to the cytoplasm in this study.

The relatively common expression of EGFRvIII (41%) was surprising but also an important finding in this study. Interestingly, in one patient we observed that while there was no detectable expression of EGFR in the primary tumour, the liver metastasis from the same patient was strongly positive for EGFRvIII expression (Data not shown). Arguably, while this observation was seen in only one patient, nevertheless it highlights an important fact that EGFR expression can alter between the primary site and distant metastatic lesions [31] and such mutations could contribute to secondary resistance to anti-EGFR mAbs therapy [40–42]. In addition, several clinical trials for vaccines targeting EGFRvIII in glioblastoma are currently underway [43]. In the first phase III immunotherapy trial (ACT IV study) with Rintega (rindopepimut) cancer vaccine, consisting of the unique 14 mer EGFRvIII peptide sequence conjugated to keyhole limpet hemocyanin, the unexpected strong performance of the control arm lead to the termination of this study although there are a subset of patients on the Rintega arm who did very well long term. It is currently unclear which factors lead to better performance in these patients. However, the high level of EGFRvIII reported in this study for patients with colorectal cancer suggest that such patients should also be included in future studies with EGFRvIII targeting vaccine.

Several studies have examined the mRNA expression levels of EGFR ligands and have found an association between the expression level of epiregulin, amphiregulin and TGFα and response to treatment with cetuximab [3, 18, 19, 24, 44, 45]. However, the increased expressed level of these genes may not always be translated into the corresponding proteins, which are directly competing with antibodies to bind on the EGFR on tumour cells. To our knowledge, there has only been one study investigating the predictive value of all EGFR ligand proteins. Yoshida and colleagues determined the expression level of EGFR ligand proteins in 26 mCRC patients with *KRAS* wild-type treated with anti-EGFR mAbs cetuximab or panitumumab. They found that co-expression of four EGFR ligands, at cut-off value of >30%, (amphiregulin, HB-EGF, TGFα, and epiregulin) might be a novel predictive biomarker of higher response rate to cetuximab and panitumumab therapy [46]. By contrast, we found the expressions of BTC and epigen to be significantly associated with a poorer OS. Since BTC is one of the most commonly expressed ligands in the gastrointestinal tract [47, 48] this could in part explain the high proportion of samples (72%) expressing BTC at a cut-off value >5%. We therefore analysed the impact of BTC expression at a cut-off value of more than 50% of the positive tumour cells on OS to minimise the effect of low specific expression of BTC. To our knowledge the association of the co-expression of wtEGFR and its ligands with clinical outcome has not been previously reported. Of note, we found the co-expressions of wtEGFR and any one ligand and in particular BTC and TGFα to be associated with poorer response to cetuximab and a shorter PFS. Indeed, analyses of the co-expression of wtEGFR and ligands higher cut-off values of >10% and >20% yielded much stronger associations with poorer response to cetuximab in this study. The abundant expression of EGFR ligands such as BTC and TGFα can result in the downregulation of the cell surface EGFR, impeding the binding ability of cetuximab, which could explain the associations with poorer response to cetuximab and shorter PFS in this study. Indeed, other studies also suggest that the cellular location of the EGFR not only can influence response to therapeutics but could also play an important role in cancer progression and patients’ survival [3, 49].

With the recent FDA approval of another anti-EGFR mAb, necitumumab [50], the undisputable fact remains that EGFR is an important therapeutic target for therapy with anti-EGFR mAbs, as well as small molecule tyrosine kinase inhibitors, albeit an expensive one. To our knowledge, the predictive value of wtEGFR in terms of its cytoplasmic location and its competing ligands and association with a shorter PFS in mCRC patients has not been previously reported. While the presence of *KRAS* and *NRAS* mutations could aid in the sparing of patients with primary resistance to treatment with anti-EGFR mAbs, it is vital to determine the relative expression and cellular location of EGFR protein (i.e. the therapeutic target) and its competing ligand proteins in patients with wild-type *KRAS* and *NRAS* prior to therapy anti-EGFR mAbs. Taken together, these could aid in the selection of a more specific population of mCRC patients who are more likely to gain long term benefit from therapy with anti-EGFR antibodies, and would spare other patients from receiving the ineffective and expensive treatment, with the associated toxicities.

Finally, in a significant number of cases, the patient has previously received post-operative adjuvant chemotherapy, before developing metastases and being considered for second time chemotherapy. Currently, most patients undergo a single assessment of their cancer's RAS status. This is often performed on a small colorectal biopsy or on a larger resection specimen of the colon or rectum, which can often pre-date the metastases by a couple of years. Less frequently, RAS assessment is performed on a recent biopsy of a metastasis or the completely resected metastatic lesion - more commonly from the lung or liver. In the former situation, the decision about using cetuximab can be based on the molecular profile of a tumour sample which may or may not be the same as that of the metastases, as seen in this study. This question of when, and from which specimen, the RAS test should be performed is something that needs further investigation, as currently it is an ad hoc process in most laboratories. As a result, large prospective randomised investigation will be vital for the validation of these findings and could lead to more effective targeted therapy of patients with anti-EGFR antibodies.

## MATERIALS AND METHODS

Ethical approval was obtained from the Research and Development Committee of the Royal Surrey County Hospital for examination of 60 mCRC surgically resected (R0) primary tumour and liver metastatic specimens from patients treated with post-operative adjuvant chemotherapy, FOLFOX or FOLFIRI plus cetuximab between May 2008 and January 2014 for use in this retrospective study. Official reports, radiographic studies, including follow up computed tomography scans were reviewed to evaluate response to cetuximab therapy using the RECIST criteria v1.1 [51].

### Immunohistochemistry

IHC staining was carried out as described previously [36], using the following primary antibodies: mouse anti-wild-type EGFR (M7298) and mouse anti-phospho-EGFR (Tyr 1173) (M7299) (Dako, UK), anti-EGFRvIII (BS-2558R, Bioss, USA), rabbit pAb anti-Amphiregulin (GTX100986, GeneTex, USA) rabbit anti-phospho-EGFR (Tyr 1068) (2234, New England Biolabs, UK), mouse anti-EGF (AHP767), mouse anti-TGFα (AHP284G) (Abd Serotec, UK), mouse anti-Betacellulin (MAB2611) (R&D Systems, UK), rabbit pAb anti-HBEGF (HPA053243), rabbit pAb anti-Epigen (HPA014420), and rabbit pAb anti-Epiregulin (HPA054373) (Sigma, UK).

### Scoring criteria

The immunostaining of the tumour sections were scored as described previously [36]. Briefly, the immunostaining of the tumour sections were scored based on the percentage of tumour cells that had HER immunostaining (i.e. > 5%, > 10%, > 20%, and > 50%) and intensity of immunostaining (i.e. negative 0, weak 1+, moderate 2+ and strong 3+) and location (i.e. membrane, cytoplasm or nucleus of the cells). Two independent observers (including a consultant histopathologist), blinded to all clinical information, conducted the scoring and any disparity in scoring was resolved by simultaneous reassessment of the staining by both observers.

### Statistical analysis

Statistical analysis was carried out in PASW statistics 22 (SPPS Inc.) using Chi-square or Fishers’ exact test (where expected counts were less than 5), Kaplan-Meier survival plots and log rank-test and Cox survival regression model. *P* ≤ 0.05 was considered statistically significant.
